# Trypanosome Lytic Factor, an Antimicrobial High-Density Lipoprotein, Ameliorates *Leishmania* Infection

**DOI:** 10.1371/journal.ppat.1000276

**Published:** 2009-01-23

**Authors:** Marie Samanovic, Maria Pilar Molina-Portela, Anne-Danielle C. Chessler, Barbara A. Burleigh, Jayne Raper

**Affiliations:** 1 Medical Parasitology, New York University Langone Medical Center, New York, New York, United States of America; 2 Immunology and Infectious Disease, Harvard School of Public Health, Boston, Massachusetts, United States of America; Washington University School of Medicine, United States of America

## Abstract

Innate immunity is the first line of defense against invading microorganisms. Trypanosome Lytic Factor (TLF) is a minor sub-fraction of human high-density lipoprotein that provides innate immunity by completely protecting humans from infection by most species of African trypanosomes, which belong to the *Kinetoplastida* order. Herein, we demonstrate the broader protective effects of human TLF, which inhibits intracellular infection by *Leishmania*, a kinetoplastid that replicates in phagolysosomes of macrophages. We show that TLF accumulates within the parasitophorous vacuole of macrophages *in vitro* and reduces the number of *Leishmania* metacyclic promastigotes, but not amastigotes. We do not detect any activation of the macrophages by TLF in the presence or absence of *Leishmania*, and therefore propose that TLF directly damages the parasite in the acidic parasitophorous vacuole. To investigate the physiological relevance of this observation, we have reconstituted lytic activity *in vivo* by generating mice that express the two main protein components of TLFs: human apolipoprotein L-I and haptoglobin-related protein. Both proteins are expressed in mice at levels equivalent to those found in humans and circulate within high-density lipoproteins. We find that TLF mice can ameliorate an infection with *Leishmania* by significantly reducing the pathogen burden. In contrast, TLF mice were not protected against infection by the kinetoplastid *Trypanosoma cruzi*, which infects many cell types and transiently passes through a phagolysosome. We conclude that TLF not only determines species specificity for African trypanosomes, but can also ameliorate an infection with *Leishmania*, while having no effect on *T. cruzi*. We propose that TLFs are a component of the innate immune system that can limit infections by their ability to selectively damage pathogens in phagolysosomes within the reticuloendothelial system.

## Author Summary

Innate immunity (present from birth) is the first line of defense against microorganisms and provides an initial barrier against disease. Here we show that a minor sub-fraction of human high-density lipoprotein (the good cholesterol), known as Trypanosome Lytic Factor (TLF), not only kills the parasite *Trypanosoma brucei*, but is also a more broadly acting antimicrobial component of the innate immune system in humans. As TLF is activated under acidic conditions, we evaluated the activity of TLF against the intracellular parasite *Leishmania*, which infects and grows within acidic compartments of macrophages, cells in our blood that normally destroy invading microorganisms. Here we show that TLF acts directly on *Leishmania* parasites, causing them to swell, thereby decreasing their infectivity. Furthermore, microscopy of macrophages infected with *Leishmania* reveal that TLF is taken up and delivered to the same compartment as *Leishmania*, concomitant with a reduction in the intracellular parasite number. Finally, we made mice that expressed the genes for human TLF; these mice reduced the pathogen burden and thereby controlled the *Leishmania* infection better than unmodified mice. In contrast, TLF mice were not protected from infection by *Trypanosoma cruzi*, a related parasite, which transiently passes through acidic compartments within cells.

## Introduction

Human blood is a potentially hostile environment to colonizing pathogens due in part to effectors of innate immunity. Trypanosome Lytic Factors (TLFs) are a subset of high-density lipoproteins (HDLs) that protect against infection by many but not all species of the African trypanosome. Two TLFs have been characterized in human blood: TLF1 and TLF2. TLF1 is a large (500 kDa) lipid rich HDL composed predominantly of apolipoprotein A-I (apoA-I), haptoglobin-related protein (Hpr), and apolipoprotein L-I (apoL-I) [1],[2]. TLF2 is a 1000 kDa lipid-poor HDL, which is an immunocomplex composed of apoA-I, Hpr, apoL-I, and IgM [1],[3]. Hpr and apoL-I are the two unique protein components of TLFs that are required to give optimal trypanolytic activity.

African trypanosomes are single cell eukaryotes (from the order *Kinetoplastida*) that live extracellularly in the bloodstream and tissue spaces of their host, from which they endocytose transferrin and lipoproteins for growth. TLF, a lipoprotein, is endocytosed by trypanosomes and trafficked to the lysosome, wherein the acidic pH activates TLF [4]–[7]. TLF forms ion selective pores in trypanosome membranes, which leads to the loss of osmoregulation allowing water influx, swelling and lysis of the trypanosomes [8],[9]. The pore forming activity has been assigned to apoL-I because a purified recombinant preparation can kill trypanosomes [9],[10]. However, *in vitro* experiments show that the association of Hpr and apoL-I in the same HDL particle is necessary to achieve optimal TLF activity, because reconstitution of individual components reveal that the combination of Hpr and apoL-I are ten-fold more lytic than either component alone and native HDLs with either Hpr or apoL-I alone have levels of activity several hundred fold lower than HDL with both Hpr and apoL-I [11]. Hpr promotes the efficient uptake of TLFs via a putative trypanosome receptor [12],[13]. The presence of an Hp (Hpr) receptor was initially reported by Drain *et al.* [13]. Recent data indicates that the trypanosome receptor ligand is in fact the complex of Hpr bound to hemoglobin (Hpr-Hb) [13],[14] and/or haptoglobin bound to hemoglobin (Hp-Hb) [15].

There are two other parasites from the order *Kinetoplastida*, *Leishmania sp.* and *Trypanosoma cruzi*, which represent important human pathogens. These parasites, which are primarily intracellular, do not have an ortholog of the Hpr-Hb receptor identified in African trypanosomes [15]. However, they do reside in an acidic parisitophorous vacuole (PV) (permanently or transiently), where TLF could be delivered, activated and act against them. We hypothesize that TLF may function more broadly as a reservoir of antimicrobial proteins such as apoL-I and Hpr-Hb that could be released from the carrier HDL and activated, in the case of *Leishmania* within the intracellular acidic PV of macrophages.

*Leishmania* is the causative agent of leishmaniasis, a disease whose manifestations in humans range from mild cutaneous and mucocutaneous lesions to fatal visceral infections. *Leishmania* undergoes a complex life cycle; human infection initiates with the deposition of non-dividing metacyclic promastigotes by sand flies biting the host skin. The parasites are then taken up by professional phagocytes [16],[17]. The major host cell is the macrophage in which the parasite resides within the PV, a phagosome that ultimately fuses with endosomes and lysosomes, forming an organelle with an acidic pH. Inside the PV, the parasites differentiate to amastigotes, multiply, and eventually rupture the cell and spread to uninfected cells [18].

*T. cruzi* is the causative agent of Chagas' disease in humans. An infected triatomine insect vector feeds on blood and deposits metacyclic trypomastigote forms of the parasite in its feces, which can enter the host through breaches in the skin or through intact mucosal membranes. *T. cruzi* trypomastigotes circulate and are disseminated to the heart and other organs through the blood, where they could encounter innate effectors. The parasite is capable of invading and replicating in a wide variety of nucleated cells in the vertebrate host. The trypomastigote form of the parasite enters a host cell and is enclosed within a membrane-delimited vacuole that rapidly fuses with lysosomes [19],[20] providing the acidic environment that is essential for vacuole disruption and parasite replication in the cytosol [21], a critical step in the *T. cruzi* life cycle.

In the present study we evaluated the effect of TLF on the intracellular growth of *Leishmania sp.* and *T. cruzi*, because they are parasites that traffic to acidified PVs to which TLF may be delivered and activated. We find that in axenic acidic conditions TLF damages metacyclic promastigotes externally and reduces their infectivity. Furthermore, TLF ameliorates infection by *Leishmania* by accumulating within the PVs of macrophages, thereby reducing the pathogen number. *In vivo*, TLF reduces the pathogen burden of *Leishmania* in mice, whereas TLF has no measurable effect on *T. cruzi* infection.

## Results

### Lytic HDL/TLF damages the metacyclic promastigotes of *Leishmania* under acidic conditions

The infective form of *Leishmania*, metacyclic promastigotes do not divide. They are covered in a dense glycocalyx of lipophosphoglycan, which contributes to their resistance to complement killing. After deposition in the skin by the bite of a sandfly, metacyclic promastigotes are rapidly opsonized and phagocytosed by macrophages and gradually fuse their PV with endosomes and lysosomes. The vacuoles containing *Leishmania* are acidified and eventually reach pH 5 [22],[23].

**Figure 1:**
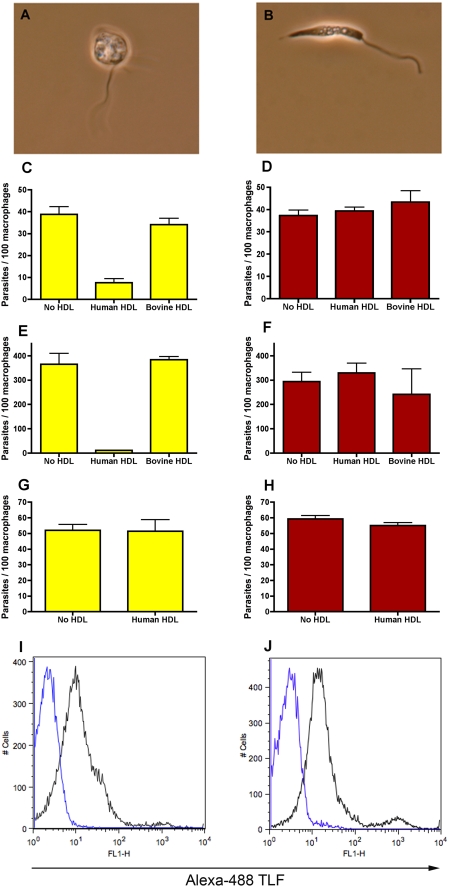
Incubation of axenic *Leishmania* promastigotes with lytic human HDL reduces the infectivity of the parasites. *L. major* metacyclics were observed under the microscope after co-incubation for 24 hours with lytic HDL (1.5 mg/ml) in acidic media pH 5.2 (A) or in neutral pH 7.4 media (B). BALB/c bone-marrow derived macrophages were infected with HDL-pre-treated *L. major* metacyclics (C,D), HDL-pre-treated *L. amazonensis* promastigotes (E,F) or HDL-pre-treated amastigote-like forms (G,H) at a multiplicity of infection of 6∶1, 10∶1, and 1∶1, respectively, for 24 hours in acidic media (yellow bars) or neutral media (red bars). The data represent the mean±standard deviation (SD) of duplicate cultures of one typical experiment that has been repeated twice. ** p<0.001 compared to bovine HDL, ANOVA test. *L. major* purified metacyclics were incubated with Alexa Fluor-488 TLF (black line) or not (blue line) for 30 min in acidic media (I) and neutral media (J), deposition of TLF was quantified by flow cytometry.

We tested the effect of lytic HDL on *L. major* and *L. amazonensis* purified metacyclic promastigotes under neutral conditions (such as those encountered in the tissues spaces and blood) and acidic conditions (such as those ultimately encountered in the PV). After 24 hours of co-incubation with a physiological concentration of lytic HDL (1.5 mg/ml), which contains TLF at physiological concentrations (∼10–15 µg/ml) at 27°C in acidic media (pH 5.2) the *L. major* metacyclic promastigotes became swollen but remained motile (Figure 1A), we could not detect any uptake of propidium iodide indicating that the parasites are still viable (data not shown). In contrast there was no visible effect of lytic HDL in neutral pH media (Figure 1B). TLF binds to the parasites independently of the pH. Incubation with Alexa Fluor-488 labeled human TLF (10 µg/ml) (pH 5.2, Figure 1I, and pH 7.5, Figure 1J) reveals a net shift in fluorescence of the whole population of parasites. Bovine HDL, which does not kill trypanosomes and does not contain TLF, was used as a non-lytic HDL control at an equivalent concentration. The parasites remained motile and elongated in acidic or neutral media in the presence of bovine HDL (data not shown).

The pretreatment of *L. major* metacyclic promastigotes with lytic HDL in acidic media substantially reduced their infectivity (p<0.01 compared to bovine HDL, Figure 1C), measured by their ability to infect BALB/c bone-marrow derived macrophages. In contrast, there was no change in infectivity after pretreatment of metacyclic promastigotes with lytic or non-lytic HDL in neutral media (Figure 1D). We observed the same outcome after pretreatment with lytic HDL of *L. amazonensis* parasites before infection of BALB/c bone-marrow derived macrophages (Figure 1E and 1F). Pretreatment of promastigotes with lytic HDL in acidic media significantly reduced their infectivity (p<0.01 compared to bovine HDL, Figure 1E). There was no change in infectivity after pretreatment in neutral media (Figure 1F). In contrast pretreatment of amastigote-like forms (day 13 of axenic transformation) with lytic HDL in acidic or neutral media did not reduce their infectivity for macrophages (Figure 1G and 1H).

We conclude that lytic HDL (which contains TLF) can damage *L. major and L. amazonensis* promastigotes under acidic conditions thereby affecting their shape and infectivity. In contrast, amastigote like forms are apparently resistant to lytic HDL.

### *L. major* encounters TLF within macrophages

Inside the macrophages, the *Leishmania* parasite resides in an acidic vesicular compartment, the PV, which has phago-endosomal/lysosomal properties. The fusion properties of the PV are dependant upon the life cycle stage used for infection *in vitro i.e.* the use of purified metacyclic promastigotes versus a heterogeneous promastigote population and the source and activation status of the host cells [23]–[28]. TLFs are a subset of HDLs and macrophages have receptors for binding and endocytosing HDLs [29]–[31] and haptoglobin [32],[33], 1% of which circulates bound to HDLs [34]. We therefore reasoned that TLF might bind to one or all of these macrophage receptors, be endocytosed, traffic to PVs and exert lytic activity against *Leishmania* at acidic pH.

**Figure 2:**
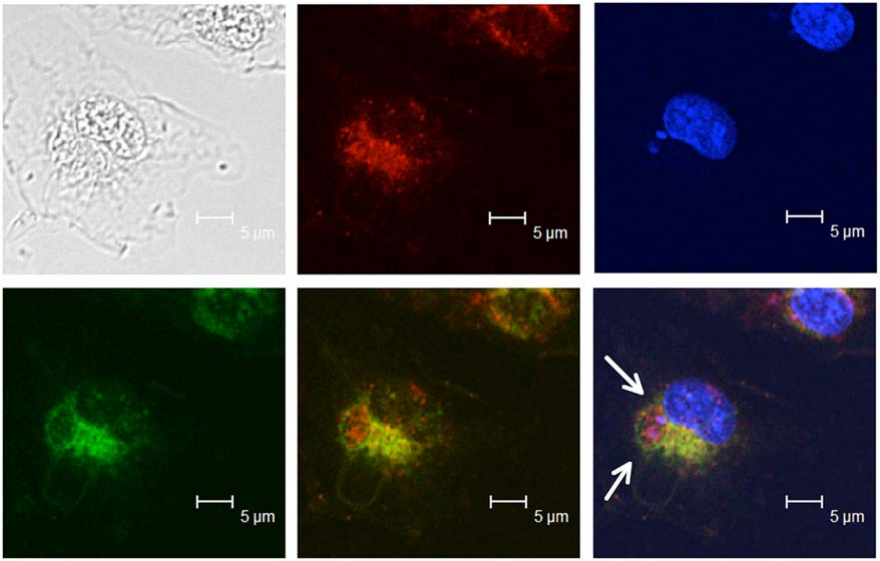
TLF accumulates within the PV in *L. major* infected macrophages. BALB/c bone-marrow derived macrophages were infected with *L. major* metacyclics for 2 hours before treatment with prelabelled-Alexa 594-TLF (10 µg/ml) for 2 hours. The grey panel is a transmission light micrograph of the imaged macrophage. The red panel depicts the uptake of labeled TLF by the macrophage. The blue panel depicts the nuclei of the macrophage (large blue area) and intracellular *Leishmania* parasites (small blue dots) stained with DAPI. Lysosomes were stained with anti-Lamp-1 antibodies (green). The merged panel shows a Lamp-1 delineated PV full of TLF (arrows). Samples were visualized with a Leica TCS SP2 AOBS confocal laser scanning microscope.

**Figure 3:**
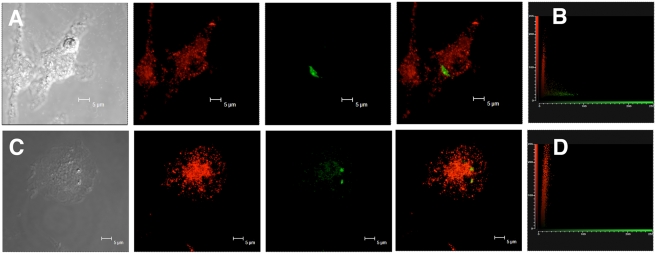
TLF surrounds parasites in the PV but is not endocytosed by *L. major*. Bone-marrow derived macrophages were infected with GFP-*L. major* parasites (green) for 2 hours before treatment with prelabelled-Alexa 594-TLF ((10 µg/ml) red) for 2 hours (A) and 24 hours (C). The grey panels are transmission light micrographs of the imaged macrophages. The lack of colocalisation of the two dyes (red and green) is revealed by the 2D cytoflurograms of 25 z-stacks (2 hours, B) and (24 hours, D). Samples were visualized and analyzed with a Leica TCS SP2 AOBS confocal laser scanning microscope.

We used confocal fluorescent microscopy to visualize the potential uptake and colocalization of TLF with *L. major* within macrophages. BALB/c bone-marrow derived macrophages were infected with *L. major* parasites for 2 hours and physiological concentrations of lytic human TLF (10 µg/ml) labeled with Alexa Fluor-594 (Figure 2). After 2 hours incubation TLF (red image) and parasites (small blue dots) were found within the phagolysosome delineated by Lamp-1 antibodies, which label all lysosomal compartments within the macrophages (green image). When all three images were merged, we observed that the parasites and TLF are found within the PV of the macrophage (Figure 2, merged panel, solid arrows). To determine whether the parasites endocytosed the TLF or were coated by the TLF within the parasitophorous vacuole we used GFP-*L. major*, which express GFP in the entire cytoplasm of the parasite. BALB/c bone-marrow derived macrophages were infected with GFP-*L. major* parasites for 2 hours. After 2 and 24 hours incubation with Alexa Fluor 594 labeled TLF there was no detectible colocalization of the two dyes, as revealed by the 2D cytofluorograms (Figure 3B, 2 h; and 3D, 24 h), which represent the data collected from 25 individual z-stacks of the two maximum projection images (Figure 3A, 2 hours; and 3C, 24 hours). Therefore, we conclude that TLF is taken up by the macrophages and surrounds the parasites within the PV but may not be endocytosed by the parasite.

### Lytic HDL/TLF kills *Leishmania in vitro* within macrophages

The fusion properties of the PV vary with the infecting species of *Leishmania*. Initially the PVs fuse with the late endosomes/lysosomes of the macrophage and eventually become fully acidified [22]–[28],[35]. Within 24 hours, *Leishmania* differentiation into amastigotes begins. *L. major* (organism of the Old World) and *L. amazonensis/L. mexicana* (of the New World) cause cutaneous leishmaniasis but diverged from each other 40–80 million years ago. Consequently, significant differences in host-parasite interactions have evolved, including differences in the PV. For example, the PVs that harbor *L. amazonensis* or *L. mexicana* (large communal PVs) versus those that harbor *L. major* or *L. donovani* (small individual PVs) indicate that the fusion/fission processes occurring at the level of these organelles differ mechanistically or kinetically in macrophages infected with these different species [35].

**Figure 4:**
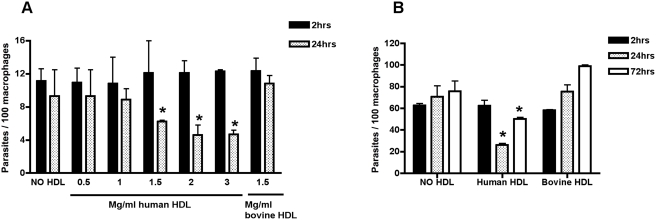
*L. major* and *L. amazonensis* are sensitive to lytic HDL within macrophages. Macrophages from Swiss-Webster intra-peritoneal cavities were infected with *L. major* metacyclics (A). BALB/c bone-marrow macrophages were infected with *L. amazonensis* promastigotes (B). The multiplicity of infection is 3∶1; parasites were incubated with macrophages for 2 hours, before the addition of human or bovine HDL at 1.5 mg/ml or at the indicated concentrations. Infected macrophages were co-incubated for 2, 24, or 72 hours. The data represent the mean±standard deviation (SD) of duplicate cultures of one typical experiment that has been repeated twice. * p<0.05 compared to bovine HDL at equivalent time points, ANOVA test.

In order to assess the effect of lytic HDL on intracellular Old World *L. major* parasites within macrophages we added different concentrations of human lytic HDL two hours post-infection of peritoneal macrophages from Swiss-Webster mice with purified metacyclic promastigotes. Bovine HDL, which does not kill trypanosomes and does not contain TLF, was used as a non-lytic HDL control. Two hours post-infection we observe an equivalent infection rate of all macrophages (Figure 4A). However, in the presence of lytic HDL the initial parasite burden of ∼11 parasites/100 macrophages was reduced to ∼5 parasites/100 macrophages (Figure 4A) after 24 hours. To evaluate the lytic capacity of HDL in large communal PVs generated by New World *Leishmania*, we repeated the 2 and 24 hours incubation with lytic HDL using BALB/c mice bone-marrow derived macrophages infected with *L. amazonensis*. Two hours post-infection we observe an equivalent infection rate of all macrophages (Figure 4B). At 24 hours *L. amazonensis* was also killed intracellularly by lytic HDL, reducing the parasite burden by ∼65% (p<0.05 compared to bovine HDL, Figure 4B). At 72 hours post-infection the parasites begin to divide within the macrophages. Of note the *Leishmania* with macrophages incubated with lytic HDL are growing at 72 hours, which suggests that the parasites have escaped the effect of lytic HDL (TLF), either by transforming or remodeling their PV or both.

**Figure 5:**
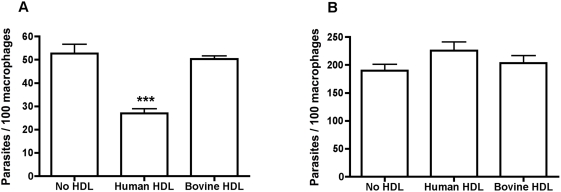
*L. amazonensis* promastigotes but not amastigotes are sensitive to lytic HDL within macrophages. BALB/c bone-marrow derived macrophages were infected with *L. amazonensis* promastigotes (A) or amastigote like forms (B) at a multiplicity of infection of 3∶1, for 2 hours before the addition of human or bovine HDL (1.5 mg/ml) for 24 hours. The data represent the mean±SD of duplicate cultures of one typical experiment that has been repeated twice. *** p<0.0001 compared to bovine HDL, ANOVA test.

Once inside macrophages metacyclics differentiate into amastigotes and begin to divide, this takes 1–3 days. We tested the susceptibility of axenically cultivated amastigote-like forms within macrophages to lytic HDL. BALB/c bone-marrow derived macrophages were infected with promastigotes (Figure 5A) or amastigote-like forms (Figure 5B) of *L. amazonensis* before treating with lytic HDL for 24 hours. There was no reduction in amastigote numbers within macrophages (Figure 5B). We conclude that amastigote-like forms of *L. amazonensis* are resistant to lytic HDL (TLF) in macrophages.

### Lytic HDL/TLF does not activate macrophages

**Figure 6:**
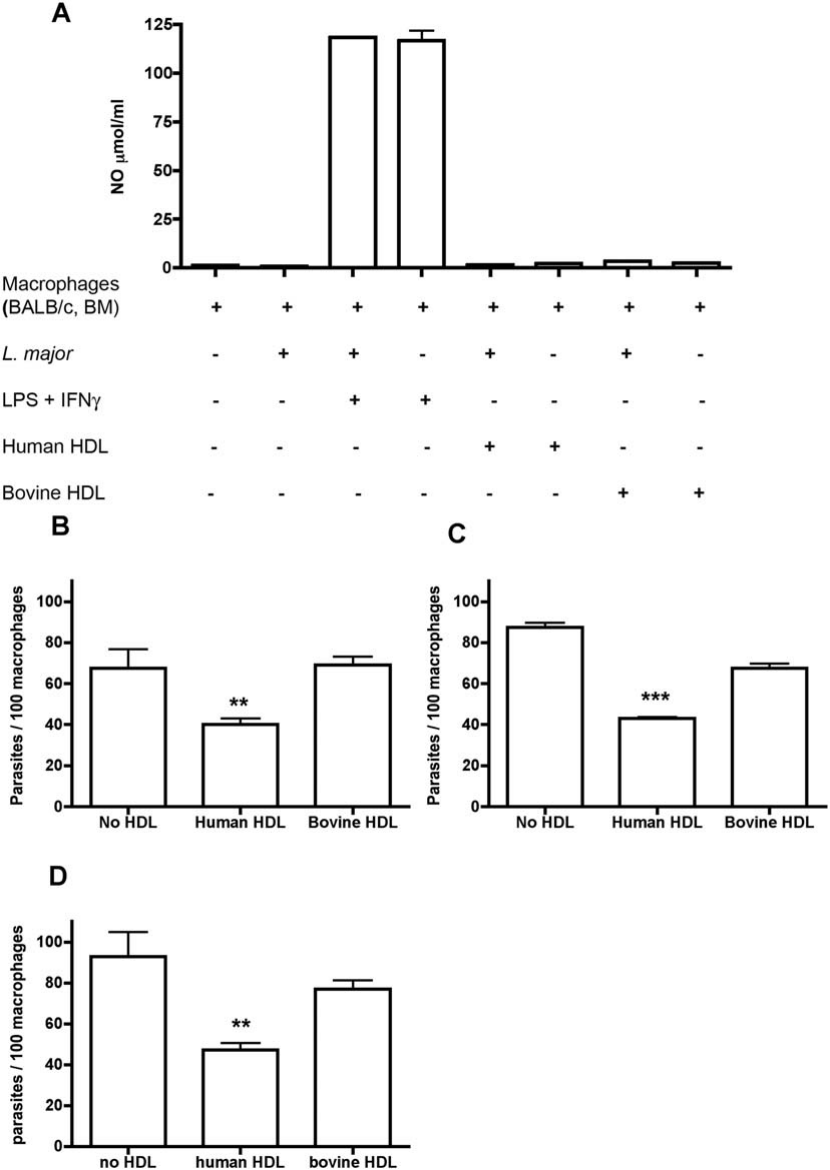
Lytic HDL does not generate NO nor requires the activation of macrophages to reduce the parasite burden. BALB/c bone-marrow derived macrophages were infected with *L. major* metacyclics at a multiplicity of infection of 3∶1, for 2 hours before the addition of bovine or human HDL (1.5 mg/ml) or treated with IFNγ and LPS; NO production in the supernatant was measured after 24 hours (A). Bone marrow derived macrophages from iNOS^−/−^ (B), gp91phox^−/−^ (C), or C57BL/6 (D) mice were infected with *L. major* metacyclics at a multiplicity of infection of 3∶1, for 2 hours, before the addition of human or bovine HDL (1.5 mg/ml). They were co-incubated for 24 hours. The data represent the mean±standard deviation (SD) of duplicate cultures of one typical experiment that has been repeated twice. ** p<0.001, *** p<0.0001 compared to bovine HDL, ANOVA test.

Binding and endocytosis of lytic human HDL (TLF) does not activate BALB/c mice bone-marrow derived macrophages infected with metacyclic promastigotes of *L. major* (Figure 6A). There was no measurable increase in nitrite oxide (NO) production unless the macrophages were treated with IFNγ and LPS. Furthermore, lytic HDL effectively reduced the parasite number in murine bone-marrow macrophages harvested from inducible NO synthase knock-out mice (iNOS^−/−^), which are unable to make NO (p<0.01, Figure 6B). Taken together the data indicate that lytic HDL does not require nor generate NO to exert anti-leishmanial activity within infected macrophages. In addition, lytic HDL effectively reduced the parasite number in murine bone-marrow macrophages harvested from NAD(P)H oxidase knock-out mice (gp91phox^−/−^) (p<0.001, Figure 6C), indicating that reactive oxygen species are not required for the anti-leishmanial activity of lytic HDL within macrophages. Lytic HDL effectively reduced the parasite number in bone-marrow macrophages harvested from the parental wild-type mice (C57BL/6 mice, p<0.01, Figure 6D). The magnitude of parasite killing in the presence of human HDL inside macrophages harvested from all three murine strains was the same (∼50%). Overall the data show that macrophages are not activated by lytic HDL and do not require activation for lytic HDL to reduce the parasite burden.

### Transgenic TLF reduces the burden of *L. major in vivo*

We next examined whether TLF can ameliorate an infection with intracellular *L. major in vivo*. Previously in our laboratory human TLF was reconstituted in transgenic mice, by generating human HDL particles that contain both apoL-I and Hpr, *in vivo* [36]. This was achieved using hydrodynamics-based gene delivery (HDG), by which single or multiple transgenes can attain a significantly high level of expression within days of DNA injection [37]. The main organs that are transfected by this technique are the liver and lungs [38]. As the liver is the main tissue that expresses the genes that encode Hpr and apoL-I (and lung), we found sufficient production and correct processing of Hpr and apoL-I occurs by this *in vivo* transfection technique [36].

**Figure 7:**
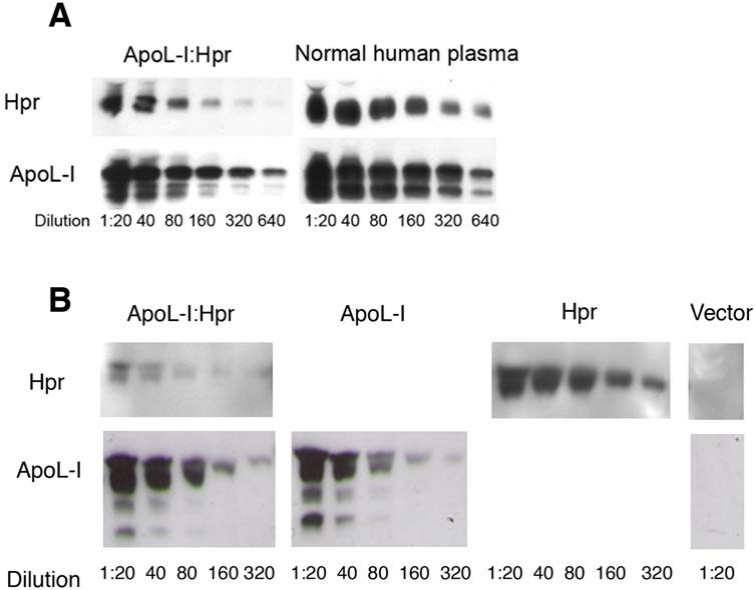
Protein levels of Hpr and apoL-I in HDG-TLF murine plasma compared to human plasma. Western blot of serial dilutions of plasma from mice expressing Hpr and apoL-I from a single plasmid (apoL-I∶Hpr) 3 days post-injection, compared to normal human plasma (A). Western blot of serial dilutions of plasma from mice injected with plasmids coding for Hpr, apoL-I, both (apoL-I∶Hpr), or no protein (vector) 3 days post-injection (B). Hpr and apoL-I were detected with monoclonal antibodies.

**Figure 8:**
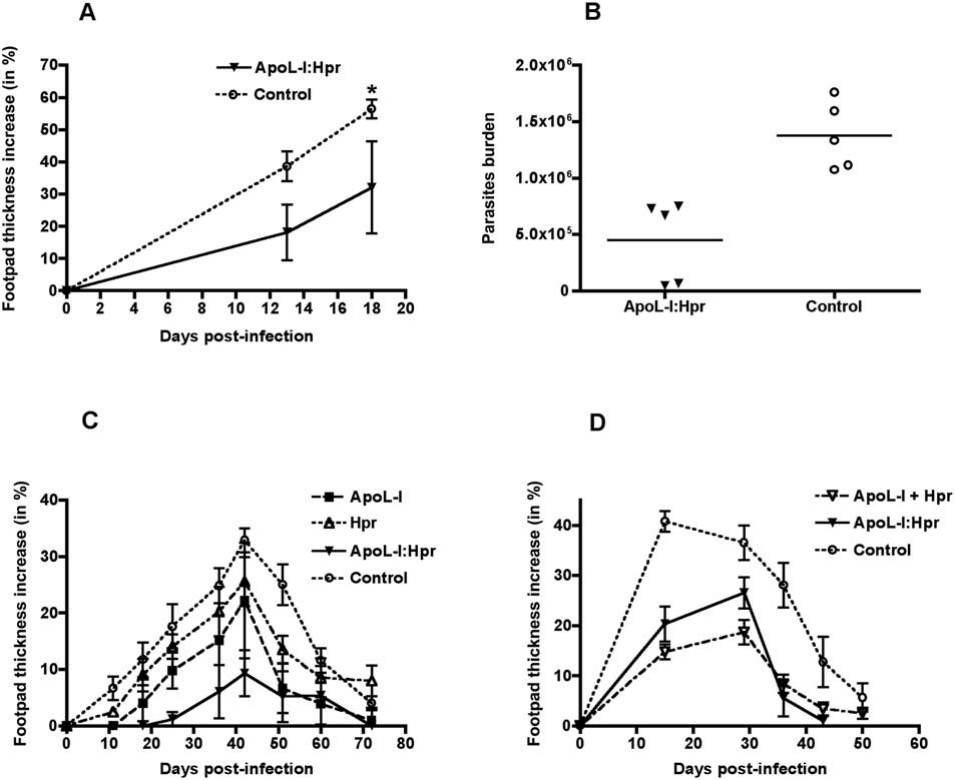
*L. major* is sensitive to transgenic lytic HDL (TLF) *in vivo*. C57BL/6 mice (5 per group) were subjected to hydrodynamic gene delivery with a single plasmid that encodes for either apoL-I, Hpr, or both apoL-I and Hpr (apoL-I∶Hpr), or two plasmids one containing Hpr and one containing apoL-I (apoL-I+Hpr) or vector alone, before subcutaneous infection of the footpad with 1×10^6^ (A,B) or 0.5×10^6^ (C,D) *L. major* metacyclics. The size of the footpad was measured with a caliper (A,C,D). (A and B) represent the data from the same experiment, in which 19 days post-infection, mice were euthanized and footpad parasites were harvested for enumeration by serial dilution assay (B). The data shown represent the mean±SD of one typical experiment that has been repeated twice. * p<0.05, Mann Whitney test.

To test the effect of reconstituted lytic HDL (TLF) on *L. major in vivo*, we transfected mice with a single plasmid that contains either apoL-I or Hpr. We also transfected mice with a single plasmid which contains both apoL-I and Hpr (apoL-I∶Hpr) under the control of individual promoters, which results in HDL particles that contain both apoL-I and Hpr [36]. We used C57BL/6 mice, which have the capacity to resolve a leishmanial footpad infection within 8–12 weeks and best “mimic” a human course of infection. ApoL-I, Hpr and apoL-I∶Hpr plasmids were injected 3 days before an *L. major* footpad infection and protein levels in the plasma and footpad size were monitored. Serial dilution of transgenic-murine plasma revealed that the level of apoL-I was approximately equivalent to that found in human plasma (Figure 7A), while Hpr was expressed at a lower level in the dual plasmid (apoL-I∶Hpr) than in the single Hpr plasmid (Figure 7B). Within 2–3 weeks post infection we observed a 50% reduction in the size of the lesion in mice expressing TLF (apoL-I∶Hpr) (p<0.05; Figure 8A), which translates into a significant three-fold reduction in parasite burden 3 weeks post-infection (p<0.05; Figure 8B).

Although HDL particles that contain both apoL-I and Hpr are more robust and have greater lytic capacity than apoL-I alone [11],[12], we have found that apoL-I is necessary and sufficient to control a trypanosome infection *in vivo* [36]. Therefore, we next investigated the individual contributions of apoL-I and Hpr toward controlling the *L. major* infection *in vivo*. In order to decrease the burden of disease and maximize the effectiveness of human TLF, the mice expressing different human TLF genes were infected with 50% fewer parasites, and the *L. major* isolate was slightly decreased in virulence by passaging one additional time *in vitro*. We found that human apoL-I (closed squares) exerted an anti-leishmanial effect that was measurable by a reduction in the footpad lesion size (p = 0.004), while the effect of Hpr (open triangle) was not significant. When Hpr and apoL-I were both expressed (closed inverted triangles) the anti-leishmanial effect appeared to be co-operative (p<0.001; Figure 8C). These results suggest that both apoL-I and Hpr are required to attain the optimal effect against *L. major* infection. Whether mice, were transfected with a single plasmid that expresses both apoL-I and Hpr (apoL-I∶Hpr), which allows for synthesis in the same transfected cell or transfected with two individual plasmids encoding apoL-I and Hpr (apoL-I+Hpr), we found that both methods of gene delivery and protein expression afford protection compared to control (Figure 8D; apoL-I∶Hpr, p = 0.045; apoL-I+Hpr, p = 0.006). Notably, complete resolution of the lesion follows a similar time course irrespective of the innate immune modulator (apoL-I alone or apoL-I and Hpr), indicating that adaptive immunity plays a key role in the resolution of the disease.

### Haptoglobin prevents lytic HDL/TLF killing of *L. major* in macrophages

**Figure 9:**
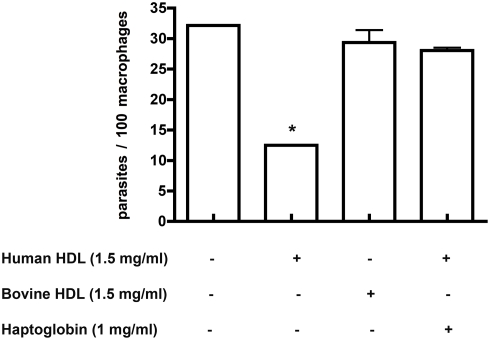
Haptoglobin inhibits lytic HDL killing of *L. major* in macrophages. BALB/c bone-marrow derived macrophages were infected with *L. major* promastigote metacyclics at a multiplicity of infection of 3∶1, for 2 hours before the addition of human or bovine HDL (1.5 mg/ml) in the presence or absence of Hp (1 mg/ml) for 24 hours. The data represent the mean±SD of duplicate cultures of one typical experiment that has been repeated twice. * p<0.05 compared to bovine HDL, ANOVA test.

In order to assess the contribution of Hpr to lytic HDL (TLF) activity on intracellular *L. major* parasites *in vivo*, we evaluated the role of Hpr as a potential ligand that facilitates the uptake and thus activity of lytic HDL in macrophages. Hp is an abundant serum protein, which when complexed with heamoglobin (Hp-Hb) is an effective inhibitor of lytic HDL (TLF) uptake into African trypanosomes [14],[15]. Therefore, we incubated BALB/c bone-marrow derived macrophages with human lytic HDL (1.5 mg/ml) for 24 hours with or without the addition of Hp (1 mg/ml) two hours post-infection with purified metacyclic promastigotes. Hp prevented lytic HDL from killing the intracellular parasites (Figure 9).

### Transgenic TLF does not affect *T. cruzi* infection *in vivo*

**Figure 10:**
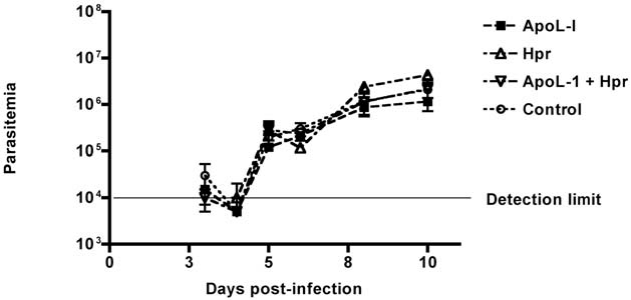
*T. cruzi* is resistant to transgenic lytic HDL (TLF) *in vivo*. Swiss-Webster mice (3 per group) injected with a control plasmid, apoL-I or Hpr plasmid alone, or both apoL-I and Hpr were infected with 1×10^6^ *T. cruzi* trypomasigotes IP three days after introduction of the genes by hydrodynamic gene delivery. The acute phase of the infection was followed by monitoring parasitemia daily. The data shown represent the mean±SD.

In order to determine if TLF would have an effect on a pathogen that transiently localizes within a phagolysosomal vacuole we compared the kinetics of infection with *T. cruzi* in wild-type mice to our TLF expressing mice. *T. cruzi* is another member of the *Kinetoplastida* that invades cells (including macrophages, smooth and striated muscle cells, and fibroblasts) passing transiently through lysosomes before escaping to the cytosol to replicate. The acute phase of infection is characterized by high blood parasitemia and tissue parasitism. Mice injected with either apoL-I or Hpr plasmid alone or both were infected three days later with *T. cruzi* trypomastigotes intraperitoneally. Expression of the apoL-I and Hpr proteins were confirmed by western blot (data not shown). The acute phase of the infection was followed by monitoring blood parasitemia daily (Figure 10). No difference in parasitemia was observed between the control mice and mice expressing apoL-I or Hpr, alone or in combination. This suggests that TLF does not have an effect on the acute stage of *T. cruzi* infection.

## Discussion

Our data shows that TLF has broad anti-microbial properties, with the ability to kill other organisms beyond trypanosomes. Because TLF requires an obligate acidic environment to become activated for pore-forming activity, we have focused on microbes that reside in an acidic environment. *Leishmania* metacyclic promastigotes are phagocytosed by macrophages wherein they transform into amastigotes within membrane-bound organelles of the endocytic pathway, progressively acquiring late endosomal/lysosomal characteristics. The phagosome acidification and fusion with the late endosomes/lysosomes is variable [22]–[28]. The differentiation to amastigotes starts in the hours following phagocytosis and takes 1 to 3 days to complete [39]. During this differentiation the parasite may be vulnerable to attack because HDL and TLF can be endocytosed and delivered to acidic endo/lysosomes in cells that have an appropriate lipoprotein scavenger receptor, such as SRB-I [29],[31] or SR-BII [30], or Hp receptors that are expressed on macrophages [32],[33].

Our results show that *L. major* parasites pretreated with lytic HDL in acidic media have a drastic change in morphology whereas in neutral media they maintained normal morphology (Figure 1A and 1B). We observed that TLF bound equally well to the parasites irrespective of the pH (Figure 1I and 1J) and that propidium iodide was excluded from the treated parasites, which suggests that the parasites remain “viable” (data not shown). However, the pretreatment of *L. major* or *L. amazonensis* promastigotes with lytic HDL in acidic media substantially reduced their infectivity; whereas, there was no change in infectivity after pretreatment in neutral media (Figure 1C–1F). We interpret this data as follows; TLF increases susceptibility to host macrophage microbicidal processes by damaging the parasites.

African trypanosomes, are killed in neutral media, because lytic HDL (TLF) is endocytosed by the parasites via a Hp-Hb receptor and activated within the acidified lysosome of the parasite, wherein it forms pores [7]–[12],[14],[15]. *Leishmania* do not have a homologue of the trypanosome Hp-Hb receptor [15] and may not be able to accumulate sufficient lytic HDL (TLF) within 24 hours. Given that the binding of TLF is equivalent in neutral or acidic media, the data suggest either (1) lytic HDL (TLF) may interact with the surface of *Leishmania* promastigotes and damage the plasma membrane when activated under acidic conditions, possibly by forming pores; or (2) TLF was endocytosed by *Leishmania* parasites and the promastigote lysosome is weakly acidified in neutral media, as it stains poorly with the pH sensitive probe, lysotracker [40], but in acidic media the parasite lysosome will be fully acidified, allowing the activation of the TLF.

**Figure 11:**
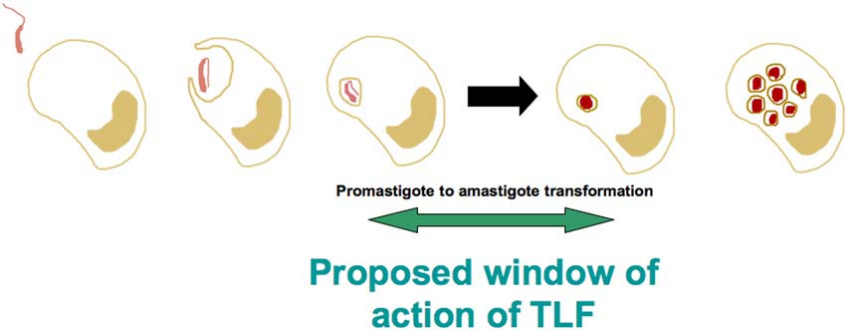
Proposed model of action of TLF against *Leishmania* parasites. The window of *Leishmania* susceptibility to lytic HDL is after phagocytosis of the metacyclic promastigotes during acidification of the PV and before transformation into amastigotes.

TLF accumulates within the PV of macrophages (Figure 2). The observation that all PVs contain TLF, which surrounds the parasites but does not appear to be endocytosed by the parasites (Figure 3), concurs with the axenic data; TLF may act directly at the parasite plasma membrane within the PV, though we cannot rule out that some TLF may be endocytosed by the parasite. We find that the number of parasites within macrophages decreased by 24 hours post-addition of lytic HDL in a dose-dependent manner (Figure 4). However, the clearance of the parasites was not complete, which may be due to individual differences each in PVs acidification process. Overall these data indicate that addition of lytic HDL (TLF) decreases the number of metacyclic promastigotes *in vitro* in macrophages. In contrast, we find that amastigotes are resistant to lytic HDL (TLF) axenically (Figure 1G and 1H) and within macrophages (Figure 5). Therefore, we conclude that the window of *Leishmania* susceptibility to lytic HDL is after phagocytosis of the metacyclic promastigotes during acidification of the PV and before transformation into amastigotes (Figure 11). Our data also show that the effect of lytic HDL on *Leishmania* is independent of macrophage activation (Figure 6).

*In vivo* infections with *L. major* lead to the development of cutaneous lesions, which are considered to arise from growth within tissue macrophages. In our *in vivo* system, apoL-I and Hpr were both required to maximally reduce the *Leishmania* lesion (Figure 8). The reduction of the *Leishmania* lesion by apoL-I was statistically significant (p = 0.004; Figure 8C). However, the dual expression of Hpr and apoL-I reduced lesion size significantly compared to apoL-I alone (p = 0.001). The reduction in lesion size was effective whether the genes were expressed from individual plasmids such that expression is from different transfected cells (p = 0.006) or the same plasmid, which allows for expression from the same transfected cell (p = 0.045; Figure 8D). Therefore, the two proteins appear to be acting co-operatively. ApoL-I likely forms a pore in the membrane of the *Leishmania* parasite directly at the plasma membrane and/or at the lysosomal membrane (Figure 1). Hpr appears to be a ligand, which binds to a putative receptor on macrophages and enhances the uptake of TLF into macrophages. We draw this conclusion from the *in vitro* competition data in Figure 9, which showed that Hp prevented the lytic HDL from killing the intracellular parasites. Recent studies demonstrate that neutrophils are the initial host cell that phagocytose a substantial fraction of *L. major* parasites after sandfly transmission [17]. Neutrophils can bind and endocytose HDLs particles [41] and Hp [42] 1% of which circulates bound to HDLs [34]. It is plausible that *in vivo*, in addition to macrophages, TLF might be endocytosed and traffic to PVs within neutrophils and exert lytic activity against *Leishmania* at acidic pH.

The co-operative effect of Hpr and apoL-I may also require Hb as proposed for African trypanosomes [14],[15]. Hb (from the FBS in culture media and in murine blood) can be bound to TLF via Hpr, and thereby be taken up by infected macrophages. The Hpr-Hb complex may be the ligand that facilitates uptake of apoL-I (in TLF complexes) into macrophages. It has been proposed that Hpr-Hb complexes may generate free radicals by reacting with hydrogen peroxide within the acidified lysosomes [14]. Although free radicals could contribute to the damage of *Leishmania* parasite membranes, we find that macrophages devoid of any NAD(P)H oxidase, which generates superoxide that can dismutate to hydrogen peroxide, are able to kill *Leishmania* (Figure 6C) as effectively as wild-type macrophages. Furthermore, Hpr-mice did not change the lesion size significantly *in vivo* (Figure 8C).

While the transgenic-TLF mice do not completely clear the *Leishmania* infection, they substantially reduce the parasitemia. The fact that *Leishmania* infection is not eliminated in the presence of TLF is not unexpected since humans have TLF but remain susceptible to *Leishmania* infection. Thus, TLF may serve to reduce the initial pathogen numbers and limit dissemination of the parasite until adaptive immunity takes effect. Other possible explanations for partial parasite clearance may be that TLF is less abundant in tissue spaces (∼25%) than in blood, and therefore TLF levels may not be optimal at the footpad lesion derma in order to act against the parasite. In addition, the hydrodynamic gene delivery system allows maximal expression of the proteins for ∼10 days. Indeed, ∼2 weeks post-injection of the plasmid, the protein expression in plasma drops below the limit of detection. Nevertheless, some low level of protein is maintained for months since mice can be infected with *T. brucei* several months post-injection of apoL-I plasmid, and resist the infection (data not shown).

In contrast to *L. major*, we could not detect any effect of transgenic-TLF against *T. cruzi* parasites (Figure 10). This finding suggests that bloodstream trypomastigotes, which accumulate to high numbers in the circulation during the acute stage of infection and invade both phagocytic and non-phagocytic cell types, are refractory to TLF. This may reflect the ability of the parasite to infect non-phagocytic cells that may not take up HDL efficiently. Additionally, it is possible that because *T. cruzi* resides transiently (8–16 hours) within acidified vacuoles, the parasites are not exposed to active TLF for a sufficient period of time.

These new findings support the hypothesis that TLF not only kills African trypanosomes, but also contributes to the innate immunity against other pathogens, such as *Leishmania*. The efficiency of killing other pathogens by TLF may depend on both a physical interaction as well as an extended period of contact between the susceptible pathogen and TLF. African trypanosomes grow in the blood and tissues spaces of the human host and constantly endocytose TLF, whereas *Leishmania* parasites grow within phagocytic cells in fully acidified PVs to which TLF may be delivered, but then transform to evade TLF action. In contrast, *T. cruzi* parasites infect non-phagocytic cells as well as professional phagocytes, and are only transiently localized within acidified vacuoles, such that constant exposure to active TLF is unlikely. We conclude that TLFs are a component of the innate immune system, which can limit infections by their ability to selectively damage pathogens such as *Leishmania*, that reside within the reticuloendothelial system.

## Materials and Methods

### Purification of human and bovine HDL

HDL was purified from normal human serum by adjusting to a density of 1.25 g/ml with potassium bromide (KBr) and ultracentrifuged at 49,000 rpm (NVTi 65; Beckman) for 16 hours at 10°C. The lipoprotein fraction was collected and the density of this fraction was adjusted to 1.3 g/ml with KBr and 4 ml aliquots were layered under 8 ml of 0.9% NaCl. The lipoproteins were then centrifuged at 49,000 rpm for 3 hours at 10°C (NVTi 65 rotor; Beckman). HDL was harvested and dialyzed against Tris-buffered saline (TBS; 50 mM Tris-HCl, 150 mM NaCl (pH 7.5) at 4°C and then concentrated by ultrafiltration (XM300 filter membrane; Amicon). HDL was concentrated to about 50 mg of protein/ml. TLF was obtained by affinity purification of human HDL using a mouse anti-human Hp monoclonal (H6395, Sigma) coupled to a HiTrap column (Amersham Biosciences). The fractions containing Hpr (TLF) were pooled and concentrated.

HDL and LDL from bovine serum cannot be efficiently separated by density, unlike human HDL and LDL. Therefore when bovine HDL was used as a control the lipoproteins were purified by adjusting their density to 1.25 g/ml with KBr and ultracentrifuged for 16 hours at 49,000 rpm, 10°C. The lipoprotein fraction (density 1–1.25 g/ml) was then collected, and size-fractionated on a Superdex 200 HR 10/30 column (Amersham) equilibrated with TBS [1]. Fractions containing apoA-I, the canonical HDL apolipoprotein, were pooled and concentrated.

### *Leishmania* preparation

*L. major* strain Friedlin V1 (MHOM/JL/80/Friedlin) and *L. major* FV1 SSU: GFP^+^(b)-SAT promastigotes were grown as previously described in medium M199 [43] (neutral medium 1), and infective-stage metacyclic promastigotes were isolated from stationary cultures (5-days old) by density centrifugation on a Ficoll gradient [44].

*L. amazonensis* IFLA/BR/67/PH8 strain promastigotes were maintained *in vitro* as previously described [45] (neutral medium 2). *L. amazonensis* axenic amastigote-like forms were cultured at 32°C in the same medium supplemented with 0.25% glucose, 0.5% trypticase, and 40 mM Na succinate (acidic medium) [45].

### *Leishmania in vitro* axenic killing assay

*L. major* and *L. amazonensis* metacyclics and amastigote-like forms were incubated for 24 hours at 27°C and 32°C respectively in corresponding neutral medium or in amastigote acidic medium in the presence or the absence of HDL. They were washed and checked for integrity under the microscope. Thereafter they were allowed to invade macrophages in DMEM containing 10% heat-inactivated FBS, 5% penicillin-streptomycin, 5 mM L-glutamine (DMEM culture medium), at a multiplicity of infection of 3 to 6 parasites per macrophage for 24 hours at 33°C (5% CO_2_, 95% air humidity). Intracellular parasites were assessed after staining with DAPI (3 µmol/L) by fluorescence microscopy.

### Macrophage preparation

Bone marrow-derived macrophages were prepared as described previously [46]. Cells were prepared from femurs of BALB/c mice (Taconic), B6;129P2-*Nos2^tm1Lau^*/J or B6.129S6-*Cybb^tm1Din^*/J, C57BL/6/J and after 3 days in culture, non-adherent progenitor cells were taken and cultured for an additional 7 days in culture medium supplemented with 30% (v/v) L cell-conditioned medium as a source of CSF-1. Adherent cells were harvested with cold DMEM+0.5 mM EDTA and seeded into an 8-well Lab-Tek II (Nalge Nunc International, Naperville, IL) chambered coverglass at a concentration of 50,000 cells/chamber and allowed to adhere for 24 hours (37°C, 5% CO_2_, 95% air humidity) before being used for infections.

Unactivated intraperitoneal macrophages were isolated by lavage of the intraperitoneal cavity of Swiss-Webster Mice (Taconic). The cells were resuspended in DMEM culture medium, seeded into an 8-well Lab-Tek II (Nalge Nunc International, Naperville, IL) chambered coverglass (50,000 cells/chamber), and allowed to adhere for 24 hours (37°C, 5% CO_2_, 95% air humidity). Thereafter, non-adherent cells were removed by three extensive washings with culture medium before being used for infections.

### *Leishmania in vitro* killing assay within macrophages

*L. major* metacyclics and *L. amazonensis* promastigotes or amastigotes were opsonized by 30 min incubation in DMEM medium containing 4% BALB/c or Swiss-Webster mouse serum and allowed to invade strain matched macrophages in DMEM culture medium, at a multiplicity of infection of 3 parasites per macrophage for 2 hours at 33°C (5% CO_2_, 95% air humidity). Thereafter, non-phagocytosed parasites were washed off, and the cultures were further incubated in the presence or the absence of HDL with or without Hp (H3536, Sigma) for indicated times. Intracellular parasites were assessed after staining with DAPI (3 µmol/L) by fluorescence microscopy.

### Assessment of nitrite oxide production by macrophages

Bone marrow-derived macrophages BALB/c mice were seeded into an 8-well Lab-Tek II chambered coverglass at a concentration of 150,000 cells/chamber before being used for infections with *L. major* at a multiplicity of infection of 3 parasites per macrophage for 2 hours at 33°C (5% CO_2_, 95% air humidity). Thereafter, non-phagocytosed parasites were washed off, and the cultures were further incubated in the presence or the absence of HDL (1.5 mg/ml) with or without murine IFNγ (5 µg/µl, 315-05, Preprotech and LPS (100 µg/µl, L6511, Sigma) for 24 hours. Nitrite quantification was measured by the Griess reaction according to the manufacturer (G7921, Molecular Probes).

### *Leishmania in vivo* inoculation and estimation of parasite load

Metacyclic promastigotes (1×10^6^) were inoculated intradermally into the right hind footpad of C57BL/6 mice (Taconic) in a volume of 50 µl using a 28.5-gauge needle (5 mice per group). The evolution of the lesion was monitored by measuring the lesion thickness with a direct-reading Vernier caliper. A non-parametric approach for several independent groups, Kuskal Wallis test, was used to analyze the data. For post-hoc comparisons, Mann Whitney tests were used with a Bonferroni correction. Parasite titrations were performed with footpad tissue homogenates obtained from individual mice and serially diluted. Each dilution was dispensed into 36 wells to give sufficient data for Poisson distribution. After 10 days, the growth of parasites was determined microscopically. The number of viable parasites in each sample was determined from the highest dilution at which promastigotes could be grown out after 7 days of incubation at 27°C. For treatment comparisons Mann Whitney tests were used.

### *In vitro* HDL/TLF and Lamp-1 staining and flow cytometry

TLFs were labeled with Alexa Fluor-594 or Alexa Fluor-488 protein labeling kit (Molecular Probes) according to the manufacturer's instructions.

*L. major* metacyclics FV1 or FV1 SUU: GFP^+^(b)-SAT purified metacyclics were opsonized by 30 min incubation in DMEM medium containing 4% serum from BALB/c mice and allowed to invade BALB/c bone-marrow derived macrophages for 2 hours at 33°C (5% CO_2_, 95% air humidity). Thereafter, non-phagocytosed parasites were washed off, and the cultures were further incubated in the presence of Alexa labeled TLF for 2 or 24 hours. Live parasites within macrophages were fixed with 2% paraformaldehyde. Cells were permeabilized with 0.05% saponin. Lamp-1 staining was performed using a rat monoclonal antibody to mouse Lamp-1 (1∶100, 1D4B; Developmental Studies Hybridoma Bank, Iowa City, IA), followed by goat anti-rat IgG conjugated to FITC antibodies (1∶200, Sigma). Intracellular parasites were observed by staining with DAPI (3 µmol/L) or direct GFP fluorescence of parasites. The samples were visualized and analyzed with a Leica TCS SP2 AOBS confocal laser scanning microscope.

For flow cytometry on live *Leishmania*, purified *L. major* metacyclics were washed twice in PBS and incubated (2×10^7^/ml) with 10 µg/ml Alexa Fluor-488 labeled TLF in bicine-buffered saline with glucose (pH 5 or 7.5) for 30 min. Cells were washed twice in FACS buffer (PBS, 5% FBS, and 0.1% sodium azide) before being analyzed. Flow cytometry was performed with a Becton Dickinson FACSCalibur system.

### Transfection of mice

Expression of human Hpr and apoL-I in plasma of mice was achieved using hydrodynamics-based gene delivery [38]. Briefly, 20 g male C57BL/6 mice (for *L. major* experiments), and Swiss-Webster (for *T. cruzi*) were injected *IV*, in less than 10 seconds with 2 ml of sterile 0.9% NaCl solution containing 50–100 µg of plasmids [36]. Three days after injections and every other day thereafter, blood samples (20 µl) were taken from the animals via tail bleeds and expression of the human proteins was evaluated by western blotting.

Plasma samples were separated on 7.5% Tris-glycine PAGE^R^ Gold precast Gels (Cambrex Bio Science Rockland, Inc. ME). Gels were transferred onto PDVF membranes (GE Healthcare Bio-Sciences, Uppsala, Sweden). For western blot analysis membranes were blocked with 5% skimmed milk and 0.1% Tween-20 in TBS and probed for 1 hour with the following antibodies: mouse monoclonal anti-Hpr (1∶5000); mouse monoclonal anti-apoL-I (1∶10,000; kindly provided by Dr. Stephen Hajduk). The secondary antibodies were conjugated to horseradish peroxidase, and used at the following dilutions: anti mouse IgG (1∶50,000; Promega, Madison, WI). Primary and secondary antibodies were diluted into 2.5% skimmed milk and 0.1% Tween–20 in TBS. Bound antibodies were detected by chemiluminescence using ECL (GE Healthcare Bio-Sciences, Uppsala, Sweden).

### *T. cruzi in vivo* inoculation and estimation of parasite load

Tissue culture-derived *T. cruzi* trypomastigotes (Y strain) were generated by weekly passage in confluent monolayers of LLcMK_2_ cells in DMEM containing 2% FBS as described previously [47]. Trypomastigotes harvested from culture supernatants were washed three times in serum free DMEM prior to use. *T. cruzi* trypomastigotes (10^6^) were injected intraperitoneally into Swiss-Webster mice (Taconic) three days after transfection (3 mice per group). Parasitemia was monitored in peripheral blood of infected mice by microscopic examination of non-fixed blood.

### Accession Numbers

Apolipoprotein L-I, NM_003661; Haptoglobin-related protein, NM_020995.

## References

[1] Lugli EB, Pouliot M, Portela Mdel P, Loomis MR, Raper J (2004). Characterization of primate trypanosome lytic factors.. Mol Biochem Parasitol.

[2] Smith AB, Esko JD, Hajduk SL (1995). Killing of trypanosomes by the human haptoglobin-related protein.. Science.

[3] Raper J, Fung R, Ghiso J, Nussenzweig V, Tomlinson S (1999). Characterization of a novel trypanosome lytic factor from human serum.. Infect Immun.

[4] Amer AO, Swanson MS (2002). A phagosome of one's own: a microbial guide to life in the macrophage.. Curr Opin Microbiol.

[5] Hager KM, Pierce MA, Moore DR, Tytler EM, Esko JD (1994). Endocytosis of a cytotoxic human high density lipoprotein results in disruption of acidic intracellular vesicles and subsequent killing of African trypanosomes.. J Cell Biol.

[6] Raper J, Nussenzweig V, Tomlinson S (1996). The main lytic factor of Trypanosoma brucei brucei in normal human serum is not high density lipoprotein.. J Exp Med.

[7] Vanhamme L, Pays E (2004). The trypanosome lytic factor of human serum and the molecular basis of sleeping sickness.. Int J Parasitol.

[8] Molina-Portela Mdel P, Lugli EB, Recio-Pinto E, Raper J (2005). Trypanosome lytic factor, a subclass of high-density lipoprotein, forms cation-selective pores in membranes.. Mol Biochem Parasitol.

[9] Perez-Morga D, Vanhollebeke B, Paturiaux-Hanocq F, Nolan DP, Lins L (2005). Apolipoprotein L-I promotes trypanosome lysis by forming pores in lysosomal membranes.. Science.

[10] Vanhamme L, Paturiaux-Hanocq F, Poelvoorde P, Nolan DP, Lins L (2003). Apolipoprotein L-I is the trypanosome lytic factor of human serum.. Nature.

[11] Shiflett AM, Bishop JR, Pahwa A, Hajduk SL (2005). Human high density lipoproteins are platforms for the assembly of multi-component innate immune complexes.. J Biol Chem.

[12] Vanhollebeke B, Nielsen MJ, Watanabe Y, Truc P, Vanhamme L (2007). Distinct roles of haptoglobin-related protein and apolipoprotein L-I in trypanolysis by human serum.. Proc Natl Acad Sci U S A.

[13] Drain J, Bishop JR, Hajduk SL (2001). Haptoglobin-related protein mediates trypanosome lytic factor binding to trypanosomes.. J Biol Chem.

[14] Widener J, Nielsen MJ, Shiflett A, Moestrup SK, Hajduk S (2007). Hemoglobin is a co-factor of human trypanosome lytic factor.. PLoS Pathog.

[15] Vanhollebeke B, De Muylder G, Nielsen MJ, Pays A, Tebabi P (2008). A haptoglobin-hemoglobin receptor conveys innate immunity to Trypanosoma brucei in humans.. Science.

[16] Rittig MG, Bogdan C (2000). Leishmania-host-cell interaction: complexities and alternative views.. Parasitol Today.

[17] Peters NC, Egen JG, Secundino N, Debrabant A, Kimblin N (2008). In vivo imaging reveals an essential role for neutrophils in leishmaniasis transmitted by sand flies.. Science.

[18] Herwaldt BL (1999). Leishmaniasis.. Lancet.

[19] Tardieux I, Webster P, Ravesloot J, Boron W, Lunn JA (1992). Lysosome recruitment and fusion are early events required for trypanosome invasion of mammalian cells.. Cell.

[20] Woolsey AM, Sunwoo L, Petersen CA, Brachmann SM, Cantley LC (2003). Novel PI 3-kinase-dependent mechanisms of trypanosome invasion and vacuole maturation.. J Cell Sci.

[21] Ley V, Robbins ES, Nussenzweig V, Andrews NW (1990). The exit of Trypanosoma cruzi from the phagosome is inhibited by raising the pH of acidic compartments.. J Exp Med.

[22] Sturgill-Koszycki S, Schlesinger PH, Chakraborty P, Haddix PL, Collins HL (1994). Lack of acidification in Mycobacterium phagosomes produced by exclusion of the vesicular proton-ATPase.. Science.

[23] Dermine JF, Scianimanico S, Prive C, Descoteaux A, Desjardins M (2000). Leishmania promastigotes require lipophosphoglycan to actively modulate the fusion properties of phagosomes at an early step of phagocytosis.. Cell Microbiol.

[24] Courret N, Frehel C, Gouhier N, Pouchelet M, Prina E (2002). Biogenesis of Leishmania-harbouring parasitophorous vacuoles following phagocytosis of the metacyclic promastigote or amastigote stages of the parasites.. J Cell Sci.

[25] Lodge R, Descoteaux A (2005). Modulation of phagolysosome biogenesis by the lipophosphoglycan of Leishmania.. Clin Immunol.

[26] Scianimanico S, Desrosiers M, Dermine JF, Meresse S, Descoteaux A (1999). Impaired recruitment of the small GTPase rab7 correlates with the inhibition of phagosome maturation by Leishmania donovani promastigotes.. Cell Microbiol.

[27] Desjardins M, Descoteaux A (1997). Inhibition of phagolysosomal biogenesis by the Leishmania lipophosphoglycan.. J Exp Med.

[28] Turco SJ, Spath GF, Beverley SM (2001). Is lipophosphoglycan a virulence factor? A surprising diversity between Leishmania species.. Trends Parasitol.

[29] Chinetti G, Gbaguidi FG, Griglio S, Mallat Z, Antonucci M (2000). CLA-1/SR-BI is expressed in atherosclerotic lesion macrophages and regulated by activators of peroxisome proliferator-activated receptors.. Circulation.

[30] Eckhardt ER, Cai L, Sun B, Webb NR, van der Westhuyzen DR (2004). High density lipoprotein uptake by scavenger receptor SR-BII.. J Biol Chem.

[31] Fluiter K, van der Westhuijzen DR, van Berkel TJ (1998). In vivo regulation of scavenger receptor BI and the selective uptake of high density lipoprotein cholesteryl esters in rat liver parenchymal and Kupffer cells.. J Biol Chem.

[32] Kristiansen M, Graversen JH, Jacobsen C, Sonne O, Hoffman HJ (2001). Identification of the haemoglobin scavenger receptor.. Nature.

[33] El Ghmati SM, Van Hoeyveld EM, Van Strijp JG, Ceuppens JL, Stevens EA (1996). Identification of haptoglobin as an alternative ligand for CD11b/CD18.. J Immunol.

[34] Kunitake ST, Carilli CT, Lau K, Protter AA, Naya-Vigne J (1994). Identification of proteins associated with apolipoprotein A-I-containing lipoproteins purified by selected-affinity immunosorption.. Biochemistry.

[35] Antoine JC, Prina E, Lang T, Courret N (1998). The biogenesis and properties of the parasitophorous vacuoles that harbour Leishmania in murine macrophages.. Trends Microbiol.

[36] Molina-Portela MP, Samanovic M, Raper J (2008). Distinct roles of apolipoprotein components within the trypanosome lytic factor complex revealed in a novel transgenic mouse model.. J Exp Med.

[37] Liu F, Song Y, Liu D (1999). Hydrodynamics-based transfection in animals by systemic administration of plasmid DNA.. Gene Ther.

[38] Kobayashi N, Nishikawa M, Hirata K, Takakura Y (2004). Hydrodynamics-based procedure involves transient hyperpermeability in the hepatic cellular membrane: implication of a nonspecific process in efficient intracellular gene delivery.. J Gene Med.

[39] Courret N, Frehel C, Prina E, Lang T, Antoine JC (2001). Kinetics of the intracellular differentiation of Leishmania amazonensis and internalization of host MHC molecules by the intermediate parasite stages.. Parasitology.

[40] Mullin KA, Foth BJ, Ilgoutz SC, Callaghan JM, Zawadzki JL (2001). Regulated degradation of an endoplasmic reticulum membrane protein in a tubular lysosome in Leishmania mexicana.. Mol Biol Cell.

[41] Shephard EG, de Beer FC, de Beer MC, Jeenah MS, Coetzee GA (1987). Neutrophil association and degradation of normal and acute-phase high-density lipoprotein 3.. Biochem J.

[42] Oh SK, Pavlotsky N, Tauber AI (1990). Specific binding of haptoglobin to human neutrophils and its functional consequences.. J Leukoc Biol.

[43] Kapler GM, Coburn CM, Beverley SM (1990). Stable transfection of the human parasite Leishmania major delineates a 30-kilobase region sufficient for extrachromosomal replication and expression.. Mol Cell Biol.

[44] Spath GF, Beverley SM (2001). A lipophosphoglycan-independent method for isolation of infective Leishmania metacyclic promastigotes by density gradient centrifugation.. Exp Parasitol.

[45] Huynh C, Sacks DL, Andrews NW (2006). A Leishmania amazonensis ZIP family iron transporter is essential for parasite replication within macrophage phagolysosomes.. J Exp Med.

[46] Tushinski RJ, Oliver IT, Guilbert LJ, Tynan PW, Warner JR (1982). Survival of mononuclear phagocytes depends on a lineage-specific growth factor that the differentiated cells selectively destroy.. Cell.

[47] Caler EV, Vaena de Avalos S, Haynes PA, Andrews NW, Burleigh BA (1998). Oligopeptidase B-dependent signaling mediates host cell invasion by Trypanosoma cruzi.. Embo J.

